# Global perspectives on the burden and management of hypophosphataemic osteomalacia in adult patients: an International Osteoporosis Foundation (IOF) survey

**DOI:** 10.1007/s11657-026-01692-y

**Published:** 2026-03-30

**Authors:** Maria Luisa Brandi, Philippe Halbout, Dominique D. Pierroz, Nicholas C. Harvey

**Affiliations:** 1 FirmoLab, FIRMO Foundation, Via San Gallo 123, Florence, 50129 Italy; 2 International Osteoporosis Foundation, Nyon, Switzerland; 3 MRC Lifecourse Epidemiology Centre, University of Southampton, Southampton, UK; 4 NIHR Southampton Biomedical Research Centre, University of Southampton and University Hospital Southampton NHS Foundation Trust, Southampton, UK

**Keywords:** Hypophosphataemic osteomalacia, Rare bone disorder, Real-world survey, Tumour-induced osteomalacia, X-linked hypophosphataemia, IOF

## Abstract

**Summary:**

This survey collected global perspectives on the burden and management of hypophosphataemic osteomalacia in adults through the International Osteoporosis Foundation (IOF). The IOF network represents a valuable international resource for understanding the burden and management of rare bone disorders.

**Purpose:**

To investigate the international burden and management of hypophosphataemic osteomalacia (HO) in adult patients.

**Methods:**

A survey was developed consisting of seven questions about respondents and their experiences of managing HO, with a second section inviting additional non-identifying information on up to five patients. The survey was disseminated to the International Osteoporosis Foundation (IOF) network.

**Results:**

Forty clinicians from 24 countries responded, with most based in academic centres. Respondents reported managing over 1000 adult patients with HO, primarily diagnosed with X-linked hypophosphataemia (XLH; 35%), tumour-induced osteomalacia (TIO; 24%), and fibrous dysplasia/McCune–Albright syndrome (FD/MAS; 16%). Management varied by diagnosis, reflecting differences in the underlying pathophysiology and clinical manifestations of the disorders. Respondents provided additional information on 19 patients with XLH, 28 with TIO, and 9 with other HO disorders. Common symptoms across XLH and TIO included bone pain (XLH, 67%; TIO, 88%), muscle pain (XLH, 61%; TIO, 76%), and muscle weakness (XLH, 61%; TIO, 88%). Many patients with XLH had discontinued phosphate and vitamin D therapies, with a subset initiating burosumab treatment. In contrast, phosphate and vitamin D were commonly used in TIO, with many patients being considered for tumour resection and limited burosumab use. Pain medication use, including opiates, was relatively high across all patients.

**Conclusion:**

Adults with HO experience a significant burden of musculoskeletal symptoms. Future efforts should focus on global education of healthcare professionals. The IOF network represents a valuable international resource for understanding the burden and management of rare bone disorders.

## Introduction

Hypophosphataemic osteomalacia (HO) is a rare metabolic bone disease characterised by chronically low serum phosphate levels, leading to impaired bone mineralisation [1]. Over time, inadequate mineralisation causes softening of the bone tissue, resulting in musculoskeletal pain and increased risk of fractures, among other symptoms [2–4]. These symptoms negatively impact all aspects of patients’ quality of life (QoL), including work, travel, physical and mental health, and social and family life [5–10]. Despite available treatment options, the rarity of HO means that most healthcare professionals (HCPs) have limited experience in managing the disease, which may lead to variations in patient care [1, 11].

Several forms of HO are mediated by fibroblast growth factor 23 (FGF23), a key regulator of phosphate homeostasis and vitamin D metabolism, which controls renal phosphate reabsorption [12].

X-linked hypophosphataemia (XLH) is the most common inherited HO disorder, with an estimated prevalence of 1.7 to 4.8 per 100,000 people [13, 14]. XLH is caused by loss-of-function mutations in the phosphate-regulating endopeptidase homolog on the X chromosome (*PHEX*) gene, leading to excessive FGF23 activity [12, 15]. Clinical features in children with XLH include rickets, lower limb deformities, delayed growth, leg deformity, bone pain, muscle weakness, fatigue, and tooth abscesses [16, 17]. Adults with XLH experience progressive functional limitations and may present with pseudofractures, osteoarthritis, enthesopathies, mobility issues, spinal stenosis, hearing loss, and dental disease [10, 16, 18].

Other inherited FGF23-mediated disorders include autosomal dominant hypophosphataemic rickets (ADHR), which results from mutations in *FGF23* itself, and autosomal recessive hypophosphataemic rickets types 1 and 2 (ARHR1/2), which are associated with mutations in dentin matrix protein 1 (*DMP1*) or ectonucleotide pyrophosphatase/phosphodiesterase 1 (*ENPP1*), respectively [1]. Additionally, fibrous dysplasia/McCune–Albright syndrome (FD/MAS) is a genetic disorder caused by a somatic gain-of-function mutation in the GNAS complex locus 1 (*GNAS1*) gene, resulting in FGF23-mediated renal phosphate wasting in approximately half of patients, a subset of whom develop HO [1, 19].

Tumour-induced osteomalacia (TIO) is a rare acquired paraneoplastic syndrome caused by excessive FGF23 secretion, typically from phosphaturic mesenchymal tumours [20]. Although fewer than 1000 cases of TIO have been reported in the literature, the true global prevalence remains uncertain [21]. Clinical features of TIO are non-specific and include bone pain, muscle weakness, and insufficiency fractures; however, nearly all cases present with hypophosphataemia, making biochemical findings a key component of diagnosis [8, 20]. Non-FGF23-mediated conditions include disorders affecting renal phosphate transport, such as Fanconi syndrome and Dent’s disease [2, 3].

Given the rarity of these disorders, most clinicians are unlikely to encounter enough cases to develop substantial clinical expertise, potentially hindering their ability to effectively diagnose and manage them [11]. This, along with regional variations in clinical practice, access to specialists, and the ability to refer to centres of excellence, may lead to inconsistent care [8, 11]. Addressing these gaps requires international insights to improve the understanding of disease burden, explore management patterns, and guide best practices. It is also important to assess whether greater disease awareness and HCP education are needed.

The long-term impact of hypophosphataemia in adults is poorly recognised and undertreated, particularly in XLH, which is often perceived as a ‘childhood’ disorder [22–24]. The rarity of these HO disorders, combined with non-specific and overlapping symptoms, often leads to delayed or missed diagnoses, increasing the risk of long-term complications and inadequate treatment in adulthood [8, 11, 23].

Given the multisystemic involvement of HO, effective management requires a multidisciplinary team (MDT) [16, 24]. This typically involves specialists such as endocrinologists, rheumatologists, nephrologists, orthopaedic surgeons, and other HCPs depending on the needs of the patient and the nature of the disorder [24]. While HO disorders share symptoms, the differences in clinical presentation should shape the MDT composition. The transition from paediatric to adult care brings further challenges, as patients experience evolving symptoms and complications [24]. Adults may struggle to access appropriate care, remaining under paediatric services or losing access to multidisciplinary support during this transition [11, 23]. It is of interest to understand how these varying aspects materialise in practice and which specialists are involved in patient care.

Management of select HO disorders has evolved with the introduction of burosumab, a fully humanised monoclonal antibody that inhibits FGF23 activity [16, 20]. Burosumab was initially approved by the US Food and Drug Administration (FDA) in 2018 for the treatment of both children and adults with XLH, and by the European Medicines Agency (EMA) for children only [25, 26]. The EMA indication expanded to include adults with XLH in 2020 [27]. That year, burosumab also received FDA approval for the treatment of TIO, followed by EMA approval in 2022 [26, 28]. Assessing the real-world management of these disorders will allow us to determine whether burosumab is being widely adopted in clinical practice.

To better understand the global management landscape of HO and address these areas of interest, an international survey was conducted among members of the International Osteoporosis Foundation (IOF) network.

### Purpose

The purpose of this study is to gather insights from HCPs involved in the diagnosis and treatment of HO and explore the global burden and management of HO in adults.

## Methods

### Survey design

The questions were developed with expert input from members of the IOF and consisted of two sections. The first included seven general questions about the participants and their experience of managing adult patients with HO. The second invited participants to provide non-identifying details on up to five patients with HO. It included six questions on each patient’s diagnosis, symptoms, current, and past treatments, along with the option to add additional details via an open-ended response. Participants were asked to provide information on at least one patient diagnosed with TIO, if they had experience managing such cases. The survey can be viewed in the Supplementary Appendix.

### Survey dissemination

The survey was hosted on SurveyMonkey^®^ and disseminated to HCPs who were members of the IOF network in January 2023 via a newsletter, dedicated emails, and social media posts (LinkedIn, Twitter [now X], and Facebook). The survey was available in English and remained open between January and March 2023. Ethical approval was not required for this study as no personal identifying information was collected, and all responses were completely anonymous. Participants were informed about the anonymous nature of the survey at the start, and no incentives or remuneration were offered for participation.

### Statistical analysis

Data were analysed using Excel and Python programming and are presented as descriptive statistics.

## Results

### Participant demographics

Forty clinicians from 24 countries completed the survey. Clinician demographics are presented in Table 1. Participants were from eight different specialties, with most being endocrinologists (40%) or rheumatologists (35%). Around two-thirds worked at academic centres (65%), one-third in hospital-based clinics (35%), and a few in private practice (15%). The number of patients under respondents’ care varied widely (between 0 and > 100), with the largest proportion (25%) caring for 1–10 patients. Three clinics in Germany, Mexico, and China had over 100 patients with HO under their care.

**Table 1:** Respondent demographics (*N* = 40)

Demographics	*n* (%)
Age, years	
20–29	3 (7.5)
30–39	5 (12.5)
40–49	9 (22.5)
50–59	7 (17.5)
60–69	11 (27.5)
≥ 70	5 (12.5)
Specialty^a^	
Endocrinology	16 (40)
Rheumatology	14 (35)
Internal medicine	4 (10)
Orthopaedic surgery	2 (5)
Geriatric medicine	1 (2.5)
Osteoporosis department	1 (2.5)
Primary care	1 (2.5)
Rehabilitation	1 (2.5)
Clinical setting^a^	
Academic medical centre	26 (65)
Hospital-based clinic	14 (35)
Large private practice group/office	4 (10)
Small or solo private practice/office	2 (5)
Primary care clinic	0 (0)
Number of patients with hypophosphataemic osteomalacia under their care (all ages)	
0	2 (5)
1–10	23 (57.5)
11–50	9 (22.5)
51–100	3 (7.5)
> 100	3 (7.5)

^a^Respondents could select more than one answer

Respondents were from France (*n* = 4), Russia (*n* = 4), Switzerland (*n* = 4), Germany (*n* = 3), the USA (*n* = 3), Argentina (*n* = 2), Italy (*n* = 2), Mexico (*n* = 2), Australia, Belarus, Belgium, Brazil, Bulgaria, Canada, China, Colombia, Egypt, Finland, India, Iran, Japan, Latvia, Spain, and the UK (*n* = 1 for each).

### Management patterns and specialty involvement in HO

**Fig. 1:**
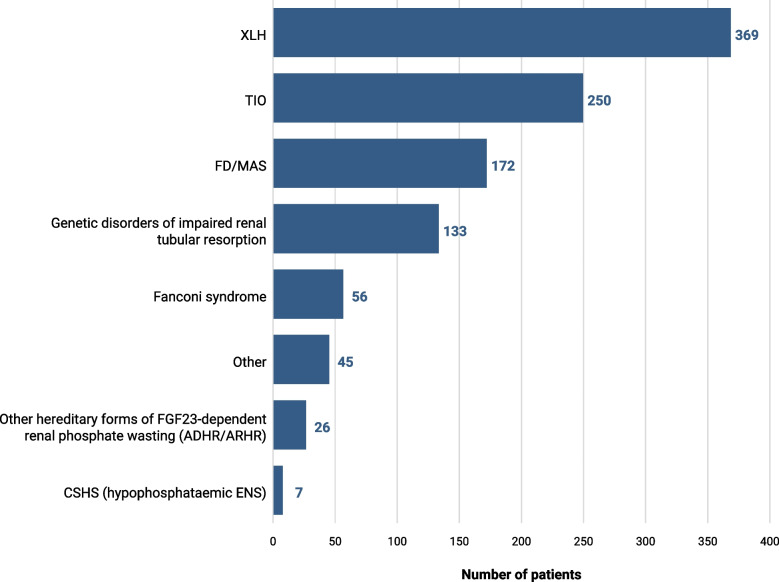
Total number of patients with HO diagnosed with each disease (*N* = 1058). *ADHR* autosomal dominant hypophosphataemic rickets, *ARHR* autosomal recessive hypophosphataemic rickets, *CSHS* cutaneous skeletal hypophosphataemia syndrome, *ENS* epidermal nevus syndrome, *FD* fibrous dysplasia*, FGF23* fibroblast growth factor 23, *MAS* McCune–Albright syndrome, *TIO* tumour-induced osteomalacia, *XLH* X-linked hypophosphataemia

**Fig. 2:**
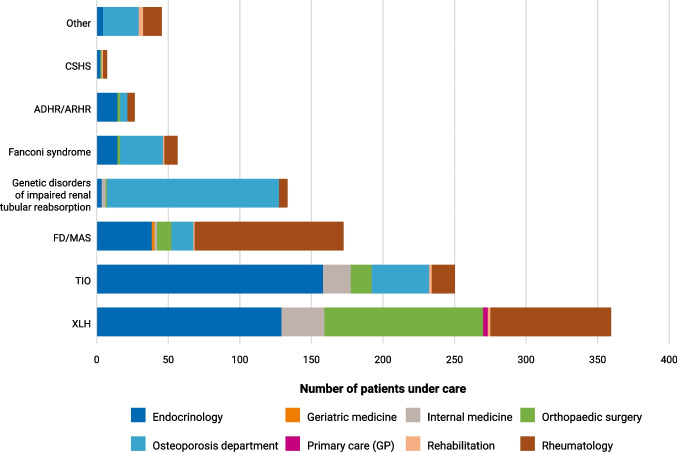
Specialties involved in the management of HO disorders (*N* = 1058). *ADHR* autosomal dominant hypophosphataemia rickets, *ARHR* autosomal recessive hypophosphataemic rickets, *CSHS* cutaneous skeletal hypophosphataemia syndrome, *FD* fibrous dysplasia, *GP* general practitioner, *HO* hypophosphataemic osteomalacia, *MAS* McCune–Albright syndrome, *TIO* tumour-induced osteomalacia, *XLH* X-linked hypophosphataemia

In the first part of the survey, respondents estimated managing over 1000 adult patients with HO, primarily diagnosed with XLH (35%), TIO (24%), and FD/MAS (16%) (Fig. 1). The remaining 25% of patients had a range of diagnoses, including genetic disorders of impaired renal tubular resorption, Fanconi syndrome, and ADHR/ARHR. Management patterns varied by diagnosis, and multiple specialties were involved in the management of each HO disorder (Fig. 2). Approximately one-third of XLH cases were managed by endocrinologists (35%) and orthopaedic surgeons (30%). TIO was primarily managed by endocrinologists (65%), while FD/MAS was more often managed by rheumatologists (60%).

### Reported burden of disease in HO

In the second section of the survey, respondents provided information on 19 adult patients with XLH, 28 with TIO, and 9 with other types of HO (FD/MAS, *n* = 3; ADHR/ARHR, *n* = 2; suspected case of TIO, *n* = 1; Fanconi syndrome, *n* = 1; drug-induced HO, *n* = 1; Raine syndrome, *n* = 1). Patient characteristics for XLH and TIO are shown in Table 2. Symptom or treatment information was not provided for one patient with XLH and three patients with TIO; these cases were excluded from further analysis (with the exception of tumour resection status or consideration for those with TIO).

**Table 2:** Patient characteristics (XLH and TIO)

**Characteristics of patients with XLH**	***N* = 19**^**a**^
Sex, *n* (%)	
Male	5 (26)
Female	14 (74)
Age, years, *n* (%)	
18–25	6 (32)
26–64	11 (58)
≥ 65	2 (11)
**Characteristics of patients with TIO**	***N* = 28**^**a**^
Sex, *n* (%)	
Male	13 (46)
Female	15 (54)
Age, years, *n* (%)	
18–25	2 (7)
26–64	23 (82)
≥ 65	3 (11)
Consideration of resection, *n* (%)	
Yes	20 (71)
No	4 (14)
Unknown	4 (14)

^a^For one patient with XLH and three with TIO, no symptom or treatment information was provided; these patients were excluded from further analysis.

TIO tumour-induced osteomalacia, XLH X-linked hypophosphataemia

**Fig. 3:**
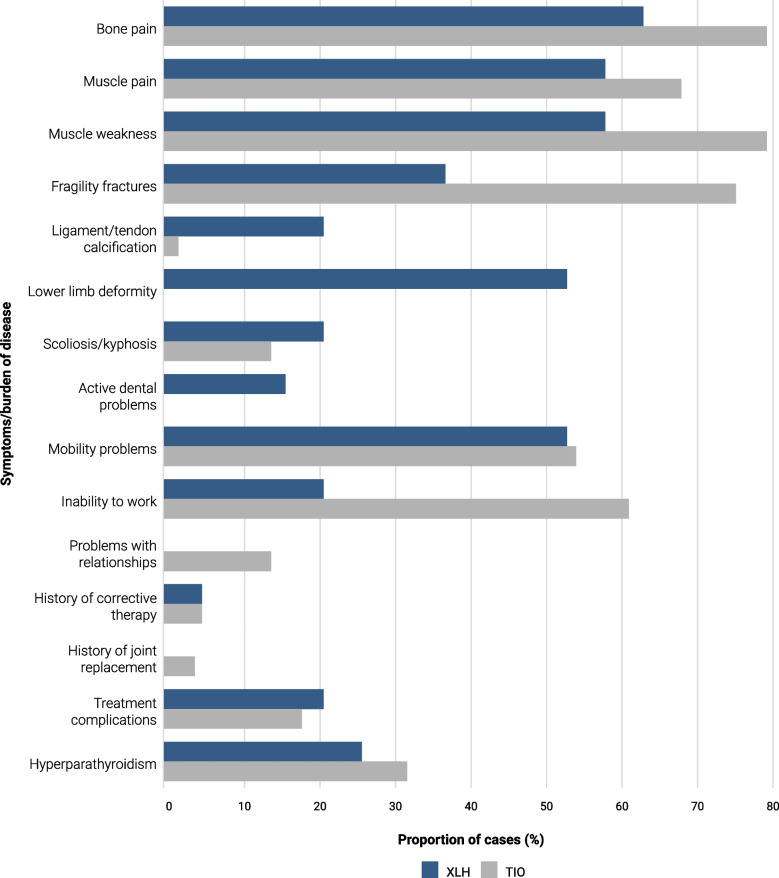
Burden of disease in patients with XLH (*N* = 18) and TIO (*N* = 25). *TIO* tumour-induced osteomalacia, *XLH* X-linked hypophosphataemia

Common symptoms across XLH (*n* = 18) and TIO (*n* = 25) included bone pain (XLH, 67%; TIO, 88%), muscle pain (XLH, 61%; TIO, 76%), and muscle weakness (XLH, 61%; TIO, 88%) (Fig. 3). More than half of the patients with XLH had lower limb deformity (56%) and mobility problems (56%), with over one-third (39%) experiencing fragility fractures^1^. The majority of patients with TIO experienced fragility fractures (84%), mobility problems (60%), and an inability to work (68%). All patients with FD/MAS (*n* = 3, 100%) reported muscle pain and weakness and were unable to work. Similarly, all patients with ADHR/ARHR (*n* = 2, 100%) experienced bone and muscle pain, as well as fragility fractures.

### Treatment patterns in patients with HO

In the second section of the survey, respondents were also asked to report the treatments they were currently prescribing and those they had previously prescribed for each patient. Among patients with XLH (*n* = 18), many had discontinued oral phosphate (previously prescribed, 72%; vs currently prescribed, 22%) and active vitamin D therapy (previously prescribed, 72%; vs currently prescribed, 39%). Over half (56%) of patients with XLH were currently receiving burosumab.

Most patients with TIO were being considered for tumour resection (*n* = 20/28, 71%), with oral phosphate and active vitamin D therapy commonly prescribed (currently prescribed in 60% and 80% of cases, respectively). Only a small number (*n* = 4, 16%) were currently receiving burosumab, with resection not being considered in three of these cases.

Pain medication use, including opiates, was relatively high across all disorders. One-third of patients with XLH (*n* = 6, 33%) were prescribed non-opiates as pain medication, while one patient (6%) received opiate medication. Over half of patients with TIO were receiving non-opiates at the time of the survey (*n* = 14, 56%) with a smaller number receiving opiates as pain medication (*n* = 3, 12%). All patients with FD/MAS (*n* = 3, 100%) and ADHR/ARHR (*n* = 2, 100%) were currently receiving non-opiates as pain medication; none was receiving opiates.

Aside from pain medication, patients with FD/MAS (*n* = 3) were prescribed a few other therapies, with two patients receiving active vitamin D therapy. Both patients with ADHR/ARHR (*n* = 2, 100%) were currently receiving phosphate supplementation, with one also receiving active vitamin D therapy. Octreotide and cinacalcet were used minimally across all disorders; each was received by one patient (4%) with TIO, and cinacalcet was received by one patient (5%) with XLH.

## Discussion

Forty respondents from 24 countries managed over 1000 adult patients with HO, suggesting that the clinicians within the IOF network collectively have considerable experience in managing these rare metabolic bone disorders. The high awareness of HO, particularly of XLH and TIO, among respondents indicates a strong global interest from specialists in this field. Three respondents from Germany, Mexico, and China reported caring for over 100 patients with HO (range 140–240). Although this figure appears high for rare disorders, these clinicians likely practice at expert reference centres receiving patients from a wide geographic area.

Clinicians treating patients with HO practice in a range of clinical settings, with academic centres being the most common. This likely reflects the role of academic centres in research, which fosters expertise and establishes them as reference centres of excellence for rare disorders such as HO. Additionally, since the survey respondents were members of the IOF network, they may have a greater involvement in research and, consequently, a higher likelihood of working in academic centres compared with other HCPs. The relatively low involvement of clinicians working in private practice (15% of respondents) is unsurprising, given the funding and insurance constraints often associated with managing these rare disorders.

Management patterns varied by diagnosis, with multiple specialties involved in the care of adults with HO, reflecting the MDT approach advocated in published recommendations and guidelines [10, 16, 29, 30]. The specialties involved generally aligned with the underlying pathophysiology and clinical manifestations for each disorder. Notably, XLH and TIO were most frequently managed by endocrinologists, consistent with the central role of altered phosphate metabolism in these disorders.

Orthopaedic surgeons were also frequently involved in the management of adults with XLH, which is consistent with literature and guideline recommendations [10, 16, 31]. Orthopaedic interventions in adults with XLH predominantly focus on addressing fractures and deformities of the lower limbs, resulting from progressive osteomalacia and prolonged weight-bearing on poorly mineralised bones and misaligned joints [10, 12, 31]. Adults with XLH also experience a high frequency and early onset of musculoskeletal conditions, including osteoarthritis, often requiring hip and knee replacement [10, 31]. Joint replacement in these patients can be challenging and should be performed by surgeons with experience in skeletal dysplasias, particularly XLH [16].

The prevalence of musculoskeletal pain and muscle weakness among adults with HO in this study is consistent with the existing literature, reinforcing the substantial burden of disease despite current treatment options [6, 9, 18, 32–34]. Although musculoskeletal symptoms were frequently reported in both patients with XLH and TIO, rates appear slightly higher among patients with TIO (Fig. 3). One possible explanation is that adults with XLH, who have experienced symptoms since childhood, may become accustomed to pain or adapt their behaviours to minimise it, which could affect symptom reporting in adulthood. Many patients also reported mobility problems, reflecting the progressive deterioration in physical function associated with HO [10, 35]. This functional decline, as well as the significant pain experienced by patients, likely contributes to the high rates of inability to work reported in the survey. These findings underscore the negative impact HO can have on multiple aspects of QoL. There is a growing recognition of the impact that the symptoms described can have on patients, and patient-important outcomes, such as musculoskeletal pain, mobility, and QoL, as they are increasingly being prioritised when assessing the efficacy of therapies [36].

Hyperparathyroidism (HPT) was reported in both patients with XLH (*n* = 5, 28%) and those with TIO (*n* = 9, 36%). HPT is a recognised complication of phosphate supplementation in XLH and can also affect patients with TIO who receive prolonged phosphate therapy, which may occur if their tumour cannot be located or resected [8, 16, 37]. The survey did not ask respondents to specify the type of HPT (primary, secondary, or tertiary), which limits the interpretation of these findings.

The apparent differences between TIO and XLH in the rates of fragility fractures (*n* = 21, 84% vs *n* = 7, 39%) and lower limb deformities (0% vs *n* = 10, 56%) are consistent with the distinct pathophysiologies of these disorders. XLH is a lifelong genetic condition, with the adult clinical presentation reflecting complications persisting from childhood and adolescence, as well as those progressively acquired in adulthood [38]. The mineral deficits associated with XLH often occur throughout skeletal development, profoundly disrupting bone modelling and leading to high rates of lower limb deformities, as reported in this survey [39]. In contrast, TIO is often acquired after growth plate closure and primarily impacts bone remodelling rather than modelling [39]. The phosphaturic mesenchymal tumours responsible for TIO have been shown to elevate FGF23 levels more than the endogenous production seen in XLH [39]. These metabolic disturbances drive increased bone turnover, leading to the high fracture burden experienced by patients with TIO [39].

The lifelong renal phosphate wasting characteristic of XLH also explains the dental problems reported in patients with XLH (*n* = 3, 17%), but not in those with TIO (*n* = 0, 0%). The frequency of dental problems in this study was lower than previously published (49–82%), possibly because these issues are managed by patients’ dentists, and their records may not have been available or shared with the clinician overseeing their XLH care [6, 31]. Dental problems, resulting from poor mineralisation of teeth and compromised periodontal tissue, are common in patients with XLH and negatively affect QoL [10]. Management of these dental complications remains an unmet need in adults with XLH, with rates of surgical interventions such as tooth extractions, root canal surgery, and dental implant procedures increasing with age; complete dental clearance in young adults is not uncommon [22, 31]. Raising awareness of XLH among dentists and integrating them into the MDT should be a priority [10].

When respondents were asked to compare the therapies previously received by patients with those received at the time of the questionnaire, more than half of patients with XLH (56%) were currently receiving burosumab, while the use of oral phosphate supplements and active vitamin D had declined. It is interesting to see the real-world uptake of burosumab in adult patients, as current evidence suggests that it may alleviate bone pain and stiffness and promote fracture healing to a degree not achieved with oral phosphate supplements and active vitamin D [16, 40, 41]. As the survey only captured whether therapies were prescribed in the past or at present, the exact timing of burosumab initiation is unclear. However, given its approval for the treatment of adults by the FDA in 2018 and the EMA in 2020, and that a substantial proportion of patients in this survey were aged ≥ 26 years (68%), we can infer that at least some patients initiated burosumab treatment during adulthood, rather than transitioning into adult care already receiving it [26, 27]. The use of burosumab was likely influenced by its availability. Local recommendations and reimbursement policies differ, and in many countries, burosumab is available for children but not adults [42]. Ongoing updates to these recommendations and policies mean that access for adults continues to change.

In contrast, few patients with TIO were receiving burosumab, likely because it is only approved by the EMA and FDA for unlocalised and unresectable cases [26, 27]. While TIO can be cured by complete surgical resection, this may not always be possible if the tumour is anatomically difficult to resect or cannot be localised, as may have been the case for the patient with suspected TIO reported in this survey [8, 29]. In such cases, prioritising access to medical treatment is essential, so it is promising that three of the four patients with TIO not being considered for resection in this survey were receiving burosumab [29]. Management is particularly challenging when the tumour cannot be localised, as radiotherapy or ablation techniques are only an option for localised, unresectable tumours [8, 29]. In our clinical experience, without local interventions to reduce FGF23 production, levels may increase to an extent where burosumab is unable to neutralise excess FGF23, potentially limiting its therapeutic effect. Additionally, TIO may recur or persist following incomplete or unsuccessful surgical resection—in which case, medical therapy should be provided [29].

Furthermore, pain management was a key component of management needed across all HO disorders. The relatively high use of pain medications, including opiates, highlights the ongoing challenges in managing chronic pain, which has been shown to negatively impact QoL [43]. Despite its profound effect on patients’ wellbeing, musculoskeletal pain in HO is often overlooked and needs to be addressed.

### Strengths and limitations

This study has some strengths; in particular, it collected responses from a diverse, international group of clinicians, providing broad perspectives on the management of HO. However, it is important to note the study’s weaknesses. As the survey asked respondents to submit patient cases, only clinicians managing patients with HO responded. This may explain the low response rate and could have led to the overrepresentation of clinicians with specialised knowledge of HO, introducing recruitment bias and limiting the generalisability of the findings to the broader clinical community. Furthermore, the requirement to review patient records may have deterred clinicians from participating. The limited number of respondents from each country prevented meaningful comparisons of management practices across regions, with most respondents based in Europe. Additionally, the small sample size meant that the findings could not be statistically validated and should be interpreted accordingly.

The survey relied on self-reported clinical practice, particularly in the second section that focused on recalling specific cases. This may have introduced recall and selection bias, as clinicians may have been more likely to report cases they see more frequently or those with more severe disease, potentially skewing findings on disease burden. In addition, symptom burden was based on clinician reporting, with no information on how symptoms such as bone pain, muscle pain, or muscle weakness were assessed. Further limitations include the small number of cases reported for certain disorders, such as FD/MAS (*n* = 3) and ADHR/ARHR (*n* = 2), limiting our ability to draw firm conclusions about management patterns or the disease burden associated with these disorders. This section also captured treatments currently or previously prescribed, but did not collect data on the treatment timings or dosages. This is particularly relevant for pain medications, as the absence of dosage information means we cannot determine whether a patient remained on treatment but at a lower dose.

This survey was also initially intended to collect information regarding the global prevalence of HO disorders; however, no robust conclusions can be drawn from these data. Future studies employing more rigorous, population-based methods are needed to estimate the global prevalence of these rare disorders.

While this survey has its limitations, key learnings can be applied to improve the design and distribution of future surveys.

### Future perspectives

There is a need for ongoing education and awareness efforts to increase clinician knowledge of HO and its management, particularly in primary care settings and in regions lacking rare bone disease experts. This will help to ensure that patients worldwide have access to optimal management. Future research should prioritise addressing the underlying pathophysiology of HO and the effective management of symptoms most commonly experienced by patients, such as bone and muscle pain and muscle weakness.

## Conclusion

The rarity of HO disorders means that most HCPs have limited exposure to these conditions, making it essential to share global perspectives and experiences of their burden and management. Our study contributes to this understanding by presenting survey findings from forty clinicians across 24 countries with experience in managing HO in adults. Awareness of XLH and TIO was high among respondents, indicating strong global interest from bone specialists; however, broader education is needed to improve early diagnosis and ensure consistent management across regions and specialties.

Our findings align closely with existing literature, reinforcing the substantial musculoskeletal burden associated with these disorders and the critical role of an MDT in their management. Additionally, the survey provides real-world insights into the evolving treatment landscape, showing that burosumab is being adopted in the adult XLH population. The relatively small sample size limited the study’s ability to robustly assess regional differences in management. Future research should address this gap to improve our understanding of how clinical practice varies worldwide.

## Data Availability

The authors confirm that the data supporting the findings of this study are available within the article and its supplementary materials.

## Footnotes

1 The survey referred to ‘fatigue fractures’; however, following retrospective review, it was determined that ‘fragility fractures’ was the appropriate terminology. This has been corrected throughout.
